# Supplementary figures and images for: The cation channel mechanisms of subthreshold inward depolarizing currents in the mice VTA dopaminergic neurons and their roles in the chronic-stress-induced depression-like behavior
Source: eLife. 2024 Dec 6;12:RP88319. doi: 10.7554/eLife.88319 (PMC11623934; doi:10.7554/eLife.88319)

Figure 1 Ai

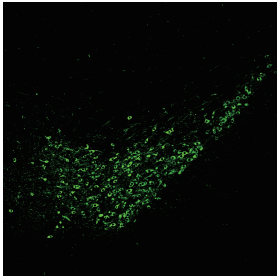

Supplement: Figure 1—source data 1. [file elife-88319-fig1-data1.pdf]

VTA-mPFC

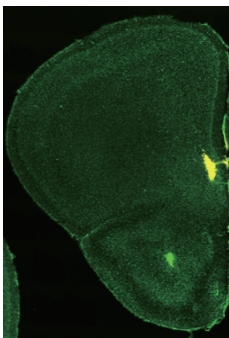

VTA-BLA

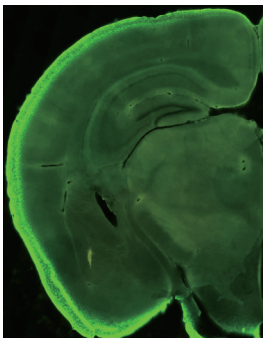

VTA-NAc ms

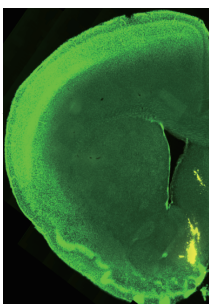

VTA-NAc c

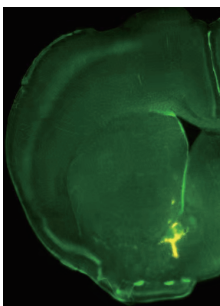

VTA-NAc ls

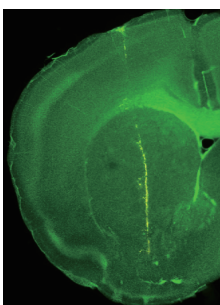

Supplement: Figure 2—figure supplement 1—source data 1. [file elife-88319-fig2-figsupp1-data1.pdf]

VTA-mPFC

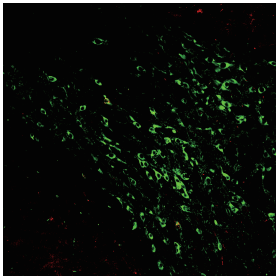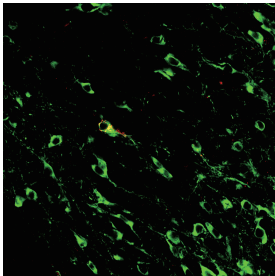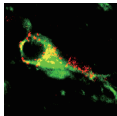

VTA-BLA

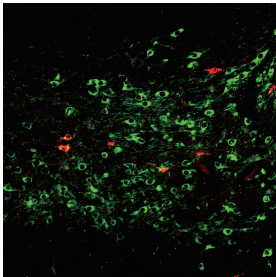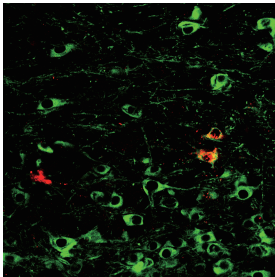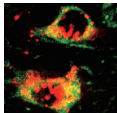

VTA-NAc ms

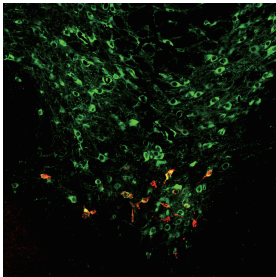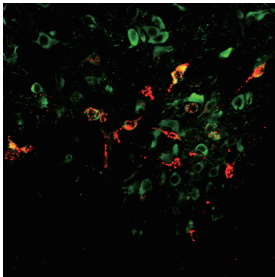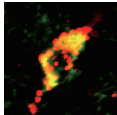

VTA-NAc c

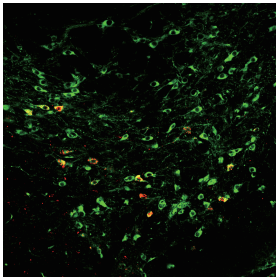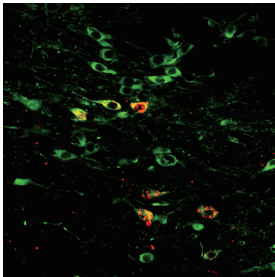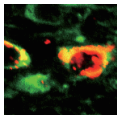

VTA-NAc ls

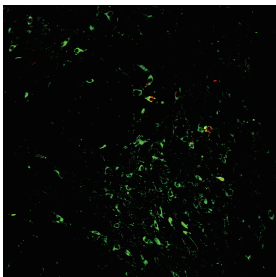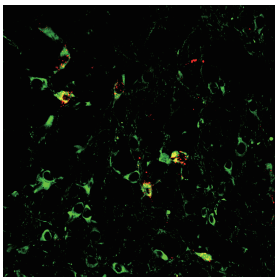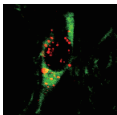

Supplement: Figure 2—figure supplement 2—source data 1. [file elife-88319-fig2-figsupp2-data1.pdf]

Figure 3 A

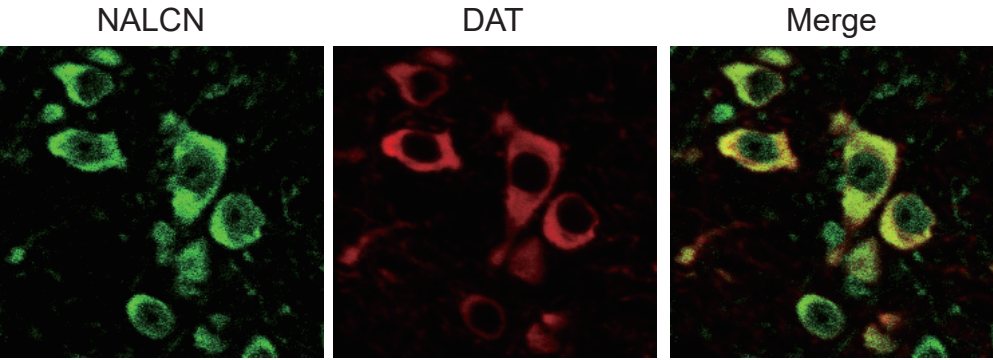

Figure 3 H

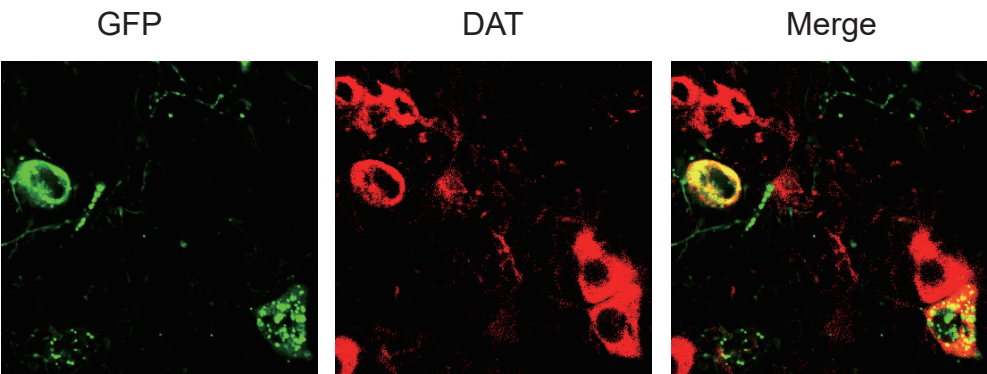

Supplement: Figure 3—source data 1. [file elife-88319-fig3-data1.pdf]

Figure 3 Bi

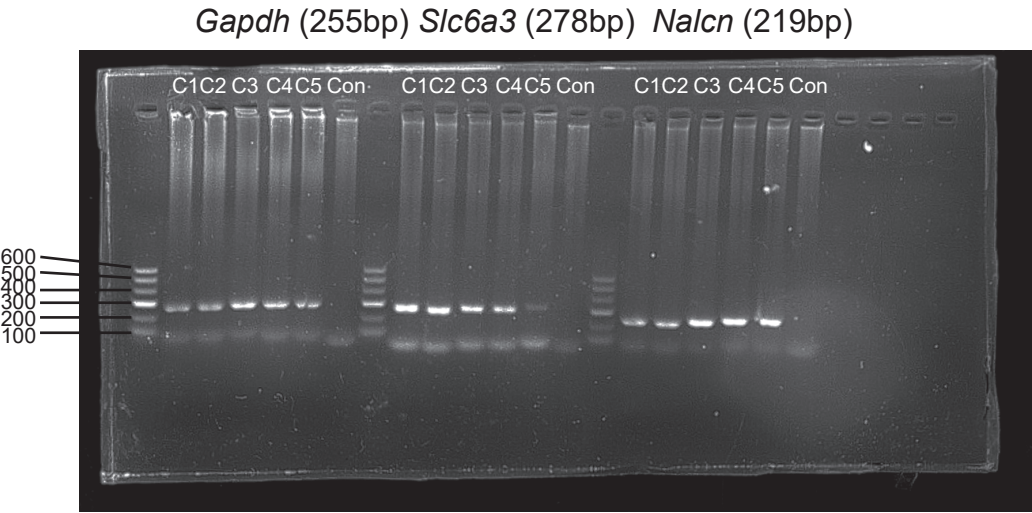

Supplement: Figure 3—source data 2. [file elife-88319-fig3-data2.zip › Figure 3 Bi_data 1/Figure 3 Bi.pdf]

Figure 4 A

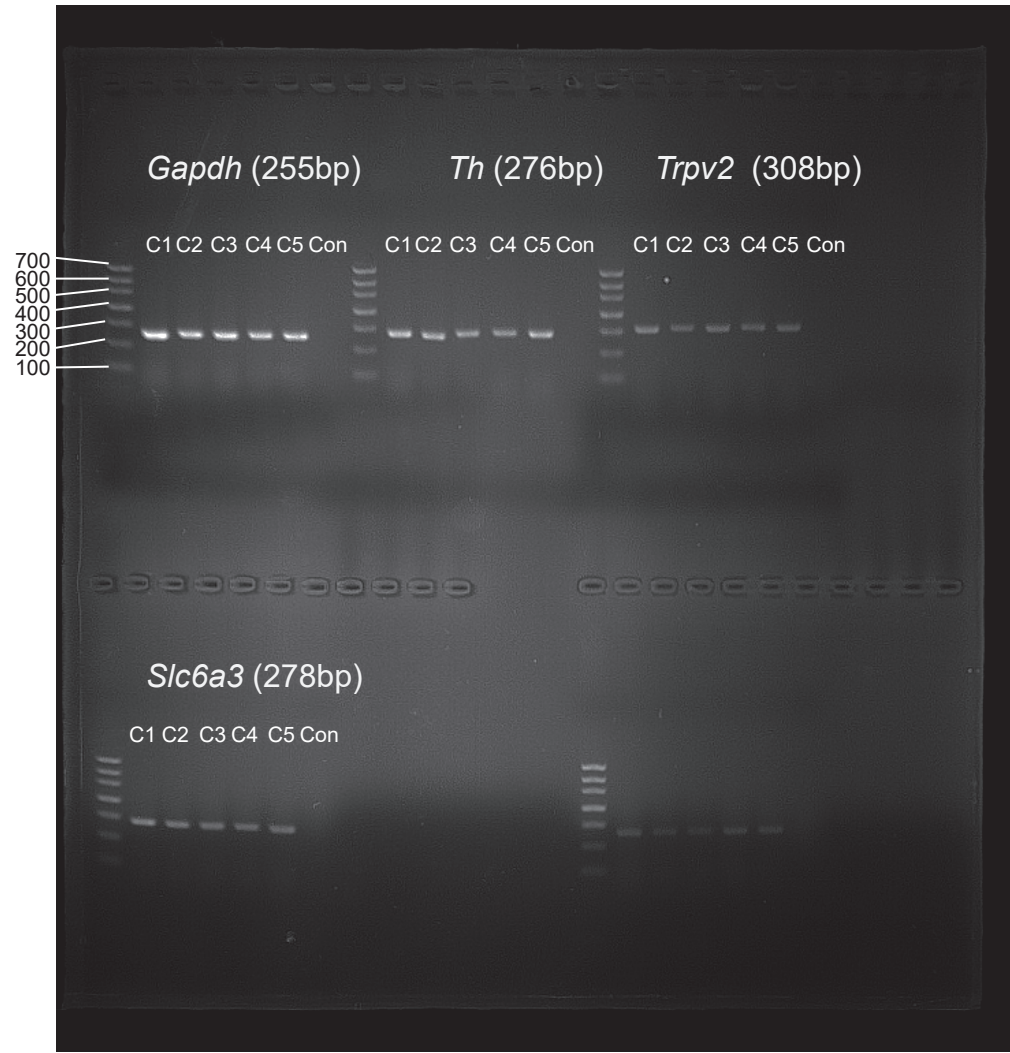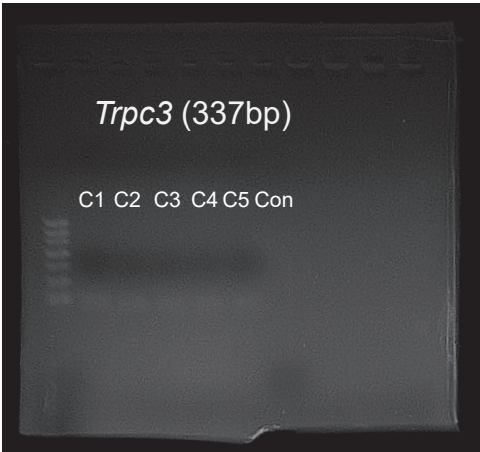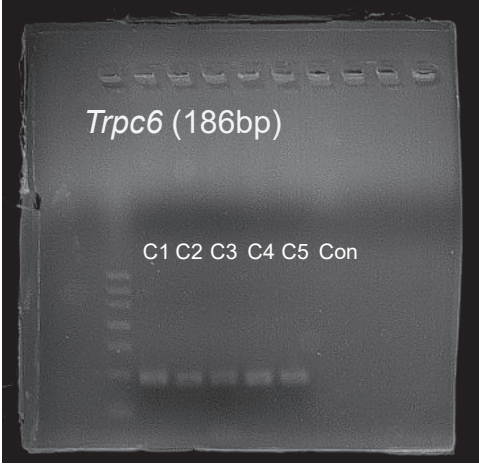

Supplement: Figure 4—source data 1. [file elife-88319-fig4-data1.zip › Figure 4 A_data 1/Figure 4 Ai.pdf]

Figure 5 B

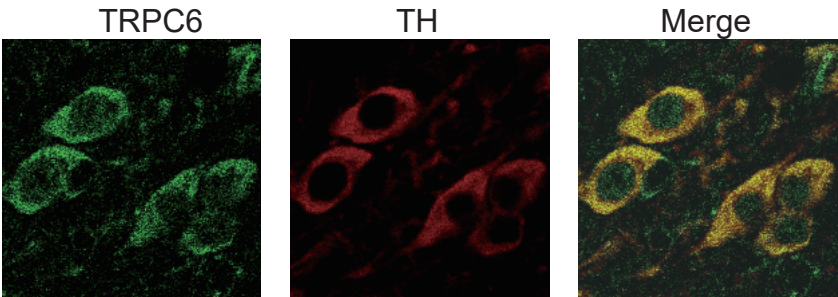

Figure 5 D

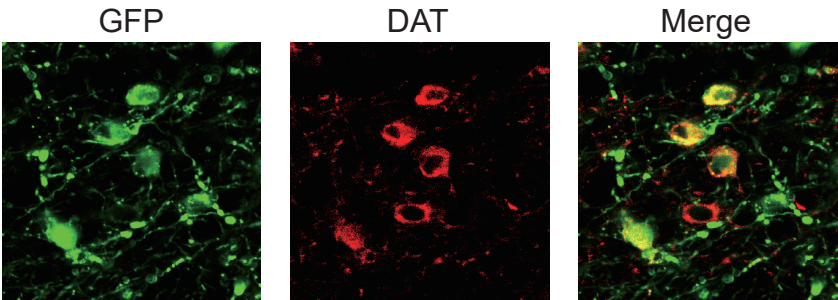

Supplement: Figure 5—source data 2. [file elife-88319-fig5-data2.pdf]

Figure 7 C

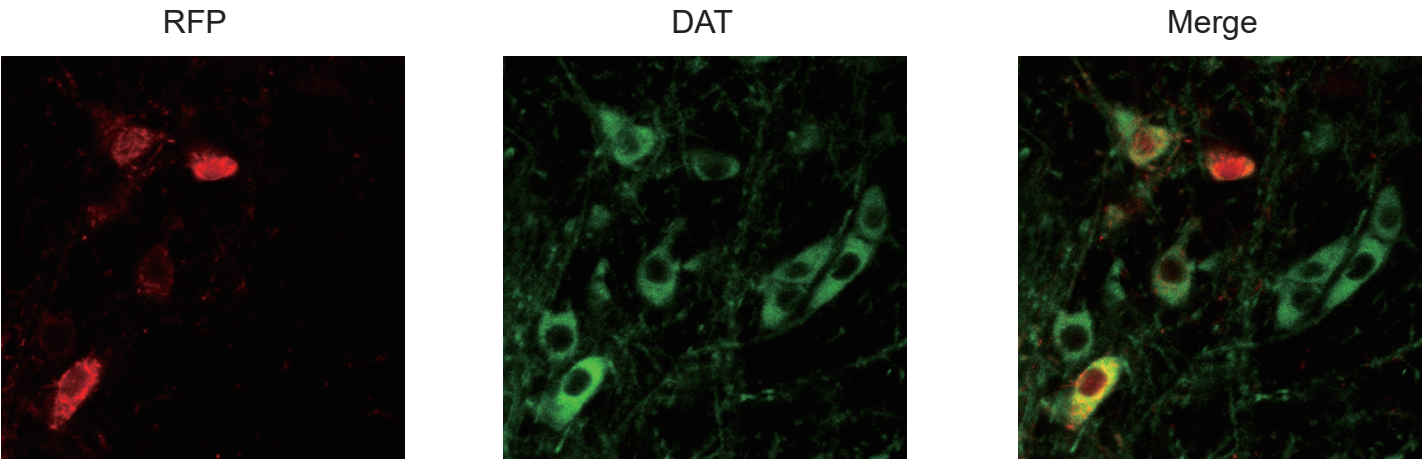

Figure 7 G

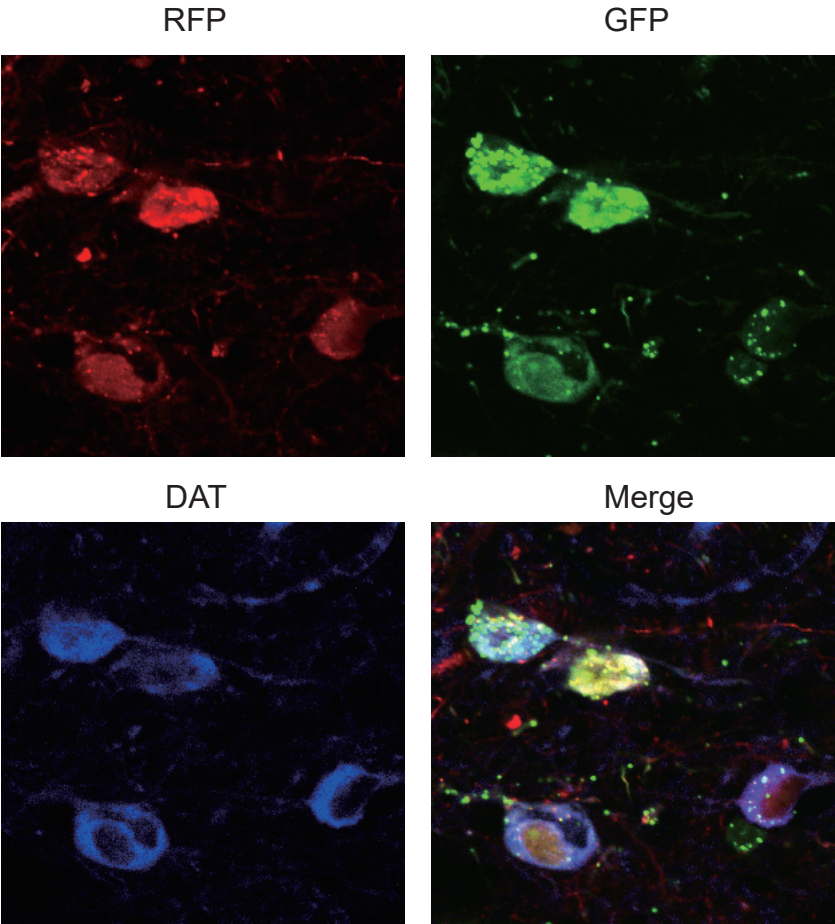

Supplement: Figure 7—source data 2. [file elife-88319-fig7-data2.pdf]

Figure 8 C

mcherry

DAT

Merge

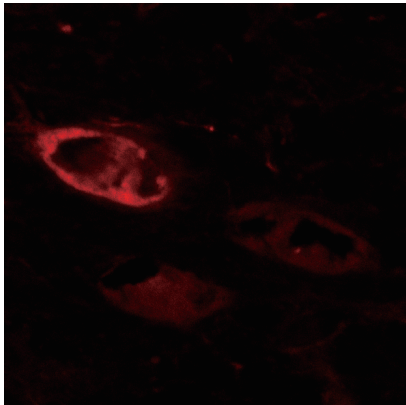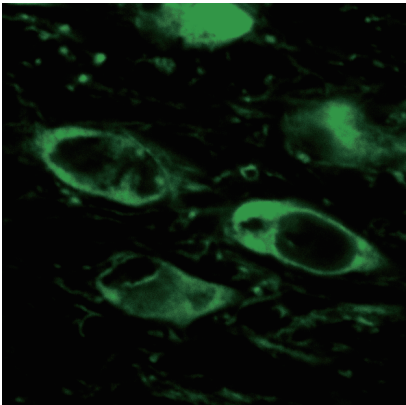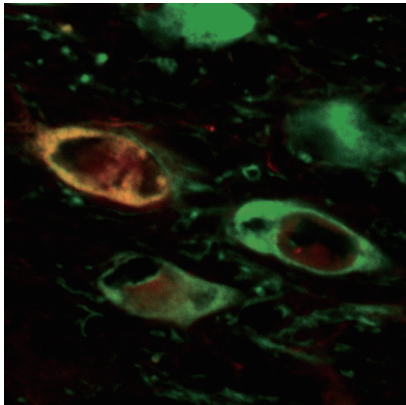

Supplement: Figure 8—source data 2. [file elife-88319-fig8-data2.pdf]
