# Supplementary material for: The cation channel mechanisms of subthreshold inward depolarizing currents in the mice VTA dopaminergic neurons and their roles in the chronic-stress-induced depression-like behavior
Source: eLife. 2024 Dec 6;12:RP88319. doi: 10.7554/eLife.88319 (PMC11623934; doi:10.7554/eLife.88319)
Supplement: Figure 5—figure supplement 1—source data 1. [file Figure 5-figure supplement 1 A.pdf › Figure 5-figure supplement 1_source data 1/Figure 5-figure supplement 1 A.pdf]

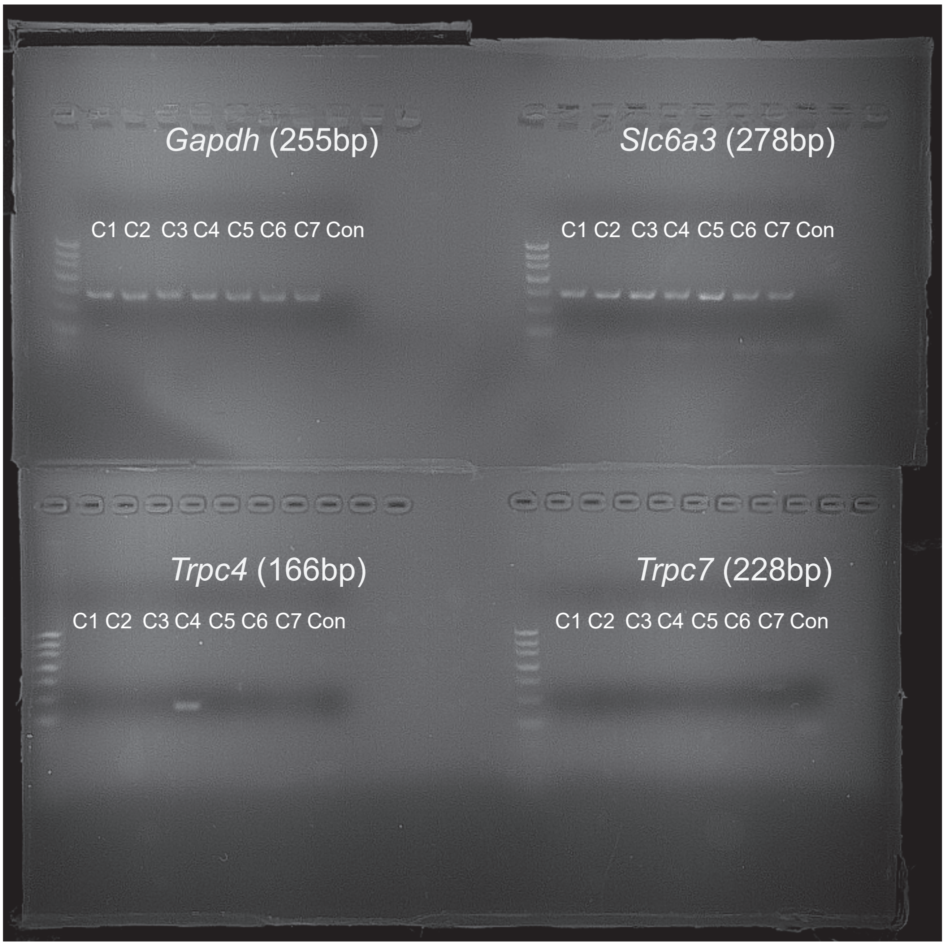
Gapdh (255bp)
C1 C2 C3 C4 C5 C6 C7 Con
Slc6a3 (278bp)
C1 C2 C3 C4 C5 C6 C7 Con
Trpc4 (166bp)
C1 C2 C3 C4 C5 C6 C7 Con
Trpc7 (228bp)
C1 C2 C3 C4 C5 C6 C7 Con

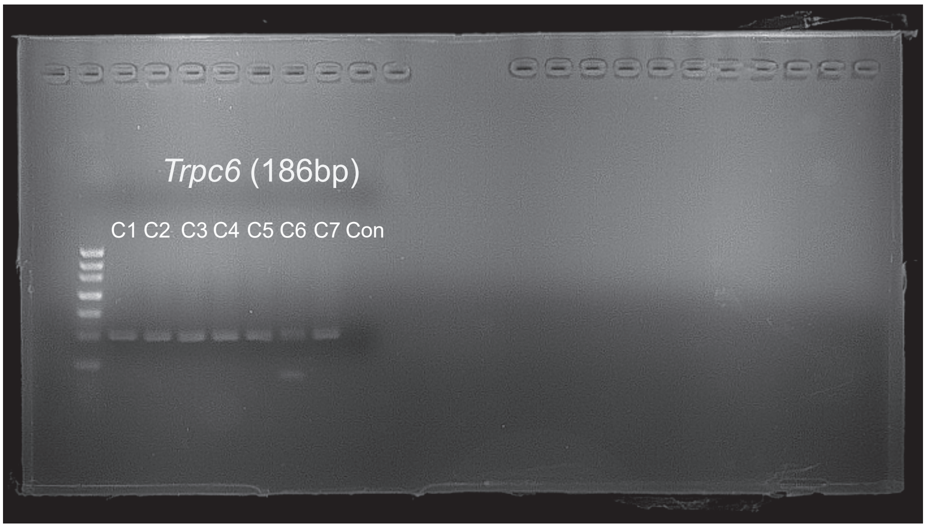
Trpc6 (186bp)
C1 C2 C3 C4 C5 C6 C7 Con
